# Voltage Imaging of Cortical Oscillations in Layer 1 with Two-Photon Microscopy

**DOI:** 10.1523/ENEURO.0274-19.2020

**Published:** 2020-05-22

**Authors:** Neil Dalphin, Kevin Dorgans, Eugene Khaskin, Bernd Kuhn

**Affiliations:** 1 Optical Neuroimaging Unit, Okinawa Institute of Science and Technology Graduate University, Okinawa 904-0495, Japan; 2 Neuronal Rhythms in Movement Unit, Okinawa Institute of Science and Technology Graduate University, Okinawa 904-0495, Japan; 3 Science and Technology Group, Okinawa Institute of Science and Technology Graduate University, Okinawa 904-0495, Japan

**Keywords:** cortex, gamma oscillation, layer 1, oscillation, two-photon, voltage imaging

## Abstract

Membrane voltage oscillations in layer 1 (L1) of primary sensory cortices might be important indicators of cortical gain control, attentional focusing, and signal integration. However, electric field recordings are hampered by the low seal resistance of electrodes close to the brain surface. To study L1 membrane voltage oscillations, we synthesized a new voltage-sensitive dye, di1-ANNINE (anellated hemicyanine)-6plus, that can diffuse into tissue. We applied it with a new surgery, leaving the dura intact but allowing injection of large quantities of staining solution, and imaged cortical membrane potential oscillations with two-photon microscopy depth-resolved (25–100 μm below dura) in anesthetized and awake mice. We found delta (0.5–4 Hz), theta (4–10 Hz), low beta (10–20 Hz), and low gamma (30–40 Hz) oscillations. All oscillations were stronger in awake animals. While the power of delta, theta, and low beta oscillations increased with depth, the power of low gamma was more constant throughout L1. These findings identify L1 as an important coordination hub for the dynamic binding process of neurons mediated by oscillations.

## Significance Statement

Here, we describe a new voltage-sensitive dye, surgery technique, and voltage measurements in cortical layer 1 (L1). The new voltage-sensitive dye di1-ANNINE (anellated hemicyanine)-6plus shows the same high sensitivity as previous ANNINE-6 dyes but diffuses better in tissue. The bubble surgery allows loading of up to 10 μl of dye or drug solution between dura and brain without dura removal or disturbance of cortical L1 in the mouse. Voltage imaging with di1-ANNINE-6plus and two-photon microscopy allows the measurement of membrane voltage spectra in L1 depth resolved up to a frequency of 50 Hz in 30 s episodes and 500 fl (1 × 5 × 100 μm^3^ line-scan volume) tissue volumes. Our measurements reveal oscillations of <20 Hz and slow gamma oscillations in the range of 35 Hz in L1 of behaving mice.

## Introduction

Cortical layer 1 (L1) is theorized to play an important role in attentional focusing (Noudoost et al., 2010), gain control (Larkum et al., 2004), and signal integration (Larkum et al., 2004). Recording from L1 using traditional neuroscience approaches has been difficult, although some advances have been made with genetic fluorescent markers (Takata and Hirase, 2008; Lacefield et al., 2019), synthetic calcium dye (Helmchen et al., 1999), and electrode recordings, plus pharmacology on the few cell types in L1 (Jiang et al., 2013, 2015; Egger et al., 2015). Dendritic tufts in L1 have been recorded with an electrode in slice preparations, but the thinness of the dendrites limits how much of the cell can be probed (Larkum et al., 2009). Additionally, L1 has been modulated through optogenetic activation of afferent fibers (Mease et al., 2016) or deactivated by optogenetic inhibition of L1 interneurons (Ibrahim et al., 2016), and the effect on other cortical areas has been measured.

Imaging neuronal activity with synthetic voltage dyes and cameras has been successfully used to elucidate functional organization of cortex (Grinvald and Hildesheim, 2004; Peterka et al., 2011), but the technique lacks depth resolution. This problem can be overcome by voltage imaging with two-photon microscopy (Kuhn et al., 2004; Fisher et al., 2005). Anellated hemicyanine (ANNINE) dyes (Hübener et al., 2003; Kuhn and Fromherz, 2003; Kuhn et al., 2004; Fromherz et al., 2008) proved to be specifically useful for voltage imaging in combination with two-photon microscopy. For a primer, see Kuhn and Roome (2019); for protocols, see Roome and Kuhn (2019b); and for a short summary, see Roome and Kuhn (2019a). If excited at the red spectral edge of absorption (Kuhn et al., 2004), ANNINE-6 dyes report linearly pure membrane voltage changes, and show large sensitivity with negligible phototoxicity and bleaching, nanosecond temporal resolution, and the possibility to combine voltage imaging with green calcium indicators like GCaMP6 (Hübener et al., 2003; Kuhn et al., 2004, 2008; Fromherz et al., 2008; Mennerick et al., 2010; Roome and Kuhn, 2018). ANNINE dyes have been bath applied (Kuhn et al., 2004), bulk loaded (Kuhn et al., 2008), or electroporated into single neurons (Roome and Kuhn, 2018) but have remained too lipophilic for loading by topical application to the brain surface. Other voltage dyes have been topically applied to the brain (Kleinfeld and Delaney, 1996; Petersen et al., 2003), or directly to the dura (Orbach et al., 1985; Lippert et al., 2007), and then left to diffuse into the upper cortical layers, thereby staining tissue. However, after removal of the topically applied dye, the dye washes out quickly from L1 (typically 100 μm thick in mice), leaving the voltage signal coming from layers 2 and 3 (Kleinfeld and Delaney, 1996; Petersen et al., 2003; Lippert et al., 2007).

We synthetized an ANNINE dye, di1-ANNINE-6plus, which diffuses better in tissue, and developed a surgery in which the dye can be applied directly to the brain surface without removing the dura. di1-ANNINE-6plus then diffuses throughout L1, allowing voltage imaging of cortical oscillations, depth resolved with two-photon microscopy.

## Materials and Methods

### 

#### Synthesis of di1-ANNINE-6plus

**Figure 1.**
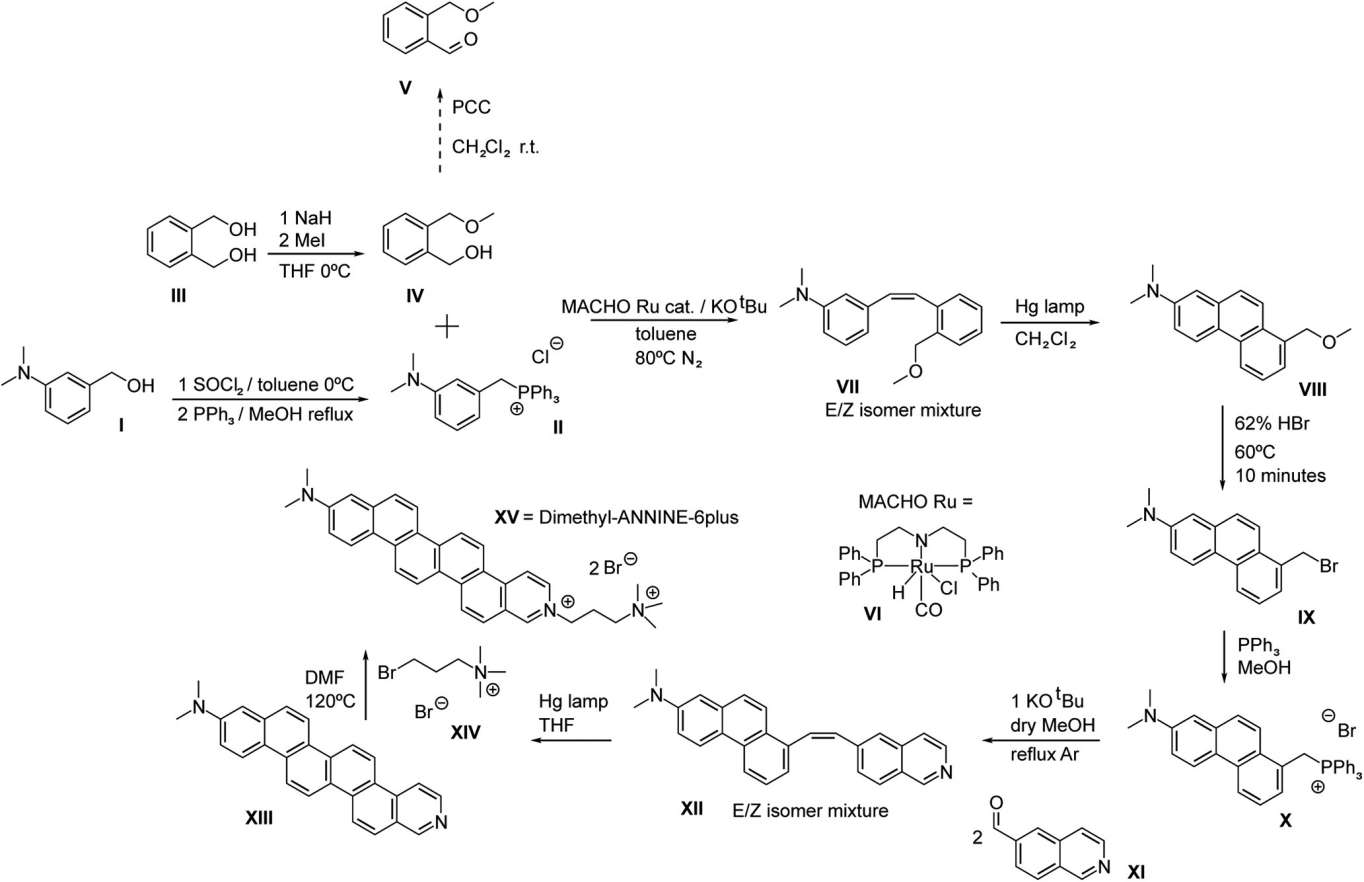
Synthesis schema for di1-ANNINE-6plus. Synthesis was adapted from Hübener et al. (2003).

The synthesis of di1-ANNINE-6plus comprises the following steps (Fig. 1).

*3-*N,N*-Dimethylaminobenzyltriphenylphosphonium chloride* (compound **II**). SOCl_2_ (39.346 g, 0.3300 mol) was slowly added to 50.00 g of alcohol [0.3300 mol 3-*N*,*N*-dimethylaminobenzyl alcohol (compound **I**); Combi-Blocks] that was earlier dissolved in toluene and cooled by an ice bath. The reaction was allowed to reach room temperature, and a precipitate formed after 1 h. The reaction was slowly quenched with concentrated NaHCO_3_ while using an ice bath until bubbling stopped. The extraction was conducted with EtOAC, and the organic layers were washed with H_2_O, NaHCO_3_, and a concentrated NaCl solution. The organics were combined, dried over MgSO_4_, filtered, and concentrated to get an oil that was mostly the chloride compound according to TLC and gas chromatography-mass spectrometry (GC-MS). Without further purification, methanol was added to dissolve the oil, and 1 equivalent of PPh_3_ (86.50 g, 0.3300 mol) was added. Afterward, the solution was heated at reflux for 1 d. After evaporating off the methanol, the remaining material was washed with 100 ml portions of diethyl ether. Eventually, the dark orange material begins to crystallize, and a light yellow solid should be obtained after enough washings. The solid is hygroscopic and, after ether evaporation under high vacuum, should be stored dry in the dark (101 g, 77.2% yield).

Note that for the subsequent reaction with alcohol catalyzed by Ru complex, it is essential to wash the Wittig salt with ether and not dichloromethane or ethyl acetate as traces of these solvents will adversely affect catalysis. If the coupling is done with the aldehyde (compound **V**), this precaution is not necessary.

*2-Methoxymethylbenzyl alcohol* (compound **IV**). A 1 l flask with dry tetrahydrofuran (THF) under argon had NaH added to it (1.74 g, 7.24E-2 mol). The temperature was lowered to 0°C and 1,2-bis(hydroxymethyl)-benzene (compound **III**; 10 g, 7.24E-2 mol; BLD Pharmatech) was added to the flask, and the mixture was stirred for a few hours at this temperature. Afterward, MeI (10.3 g = 4.51 ml, 7.24E-2 mol) was added as quickly as possible with rapid stirring. After 5 min, the reaction was quenched with water and extracted in diethyl ether and washed with water, concentrated NH_4_Cl, and brine. After drying over MgSO_4_, filtering, and concentrating, a yellow oil was obtained that contained 92% to 95% of compound **IV** in quantitative yield, with the rest being a small impurity of 1,2-dimethoxymethyl benzene. The oil was used as is without further purification in the oxidation to benzaldehyde, or the direct Ru catalyzed coupling reaction.

*E/Z-2-(3-*N,N*-dimethylaminostyryl)benzylmethyl ether* (compound **VII**). The procedure was adopted from Khaskin and Milstein (2015); however, the cheaper Ru-MACHO (compound **VI**) catalyst was used in lieu of the Milstein catalyst. Two grams g of 3-*N*,*N*-dimethylaminobenzyltriphenylphosphonium chloride (compound **II**; 4.73E-3 mol) were added to a 100 ml flask with ∼10 ml of toluene under argon equipped with a reflux condenser. KO^t^Bu (0.745 g, 4.79E-3 mol), compound **VI** (3 mg, 0.1 mol%, 4.73E-6 mol; TCI), and compound **IV** (591 mg, 5.27E-3 mol) were added subsequently and sequentially. The reaction was then refluxed overnight. After quenching with water, and extracting in EtOAc, the organic layers were washed with H_2_O, NaHCO_3_, and concentrated NaCl. The organics were combined, dried over MgSO_4_, filtered, and concentrated. The product was purified by column chromatography (9:1/EtOAc: hexanes) to get E/Z-2-(3-*N*,*N*-dimethylaminostyryl)benzylmethyl ether (compound **VII**; 1.01 g, 79.9%). Subsequently, the reaction was successfully repeated on a 20 g scale of compound **II** (30 mg catalyst); however, the yield was less (∼50%).

*7-*N,N*-dimethylamino1-methoxymethylphenanthrene* (compound **VIII**). Two grams of compound **VII** (7.48E-3 mol) were dissolved in distilled THF (200 ml) in a flask equipped with a reflux condenser under argon and irradiated by a Hg high-pressure lamp (Sun Energy) for 16 h at room temperature. The reaction could be followed by TLC and GC-MS. After conversion of the starting material, the solvent was evaporated and the product was purified by column chromatography (12:1–8:1 gradient/EtOAc: hexanes) to get 7-*N*,*N*-dimethylamino1-methoxymethylphenanthrene (compound **VIII**; 1.20 g., 60.5%) as a light yellow solid.

*E/Z-5-(7-*N,N*-Dimethylamino-1-phenanthryl)vinylisoquinoline via 7-*N,N*-dimethylaminophenanthryl-(1)-methyltriphenylphosphonium bromide* (compound **X** and **XII**) *and 7-*N,N*-dimethyllamino-1-bromomethylphenanthrene* (compound **IX**). One gram of compound **VIII** (3.77E-3 mol) was put in a small flask to which 20 ml of 62% HBr was added. The mixture was stirred at 60°C for 10 min and subsequently cooled. The mixture was neutralized with NaHCO_3_. The product was extracted with EtOAc and washed with NaHCO_3_ and concentrated NaCl. After drying over MgSO_4_, it was filtered and concentrated to get the bromide. This compound should not be stored for prolonged periods of time or cleaned on the column, as this leads to decomposition. Immediately after synthesis, the compound is ∼95% pure according to GC-MS.

Half of the bromide portion (1.88E-3 mol) was dissolved in 30 ml of methanol and PPh_3_ (0.54 g, 2.08E-3 mol) were added, and the solution was refluxed overnight. After removing MeOH under reduced pressure, the viscous orange liquid solidified after washing several times with Et_2_O and was stored in the dark.

Subsequently, the solid was redissolved in dry methanol under argon and KO^t^Bu (209 mg, 1.87E-3 mol) was added, and the solution was refluxed for 3 h. Afterward, 5-formylisoquinoline (compound **XI**; 294 mg, 1.87E-3 mol; Sigma-Aldrich) was added, and the solution was refluxed overnight. After evaporating off the solvent, the organics were extracted in CH_2_Cl_2_ and washed with water, concentrated NaHCO_3_, and concentrated NaCl, dried over MgSO_4_, filtered, and concentrated to give a brownish solid containing triphenyl phosphine oxide and E/Z-5-(7-*N*,*N*-dimethylamino-1-phenanthryl)vinylisoquinoline (compound **XII**). This solid could be washed ∼10× with Et_2_O to get a bright yellow solid that is soluble in chloroform and was determined to be pure (mixture of E/Z isomers) compound **XII** by nuclear magnetic resonance (NMR) and electrospray ionization MS (0.25 g., 36% yield).

*11-*N,N*-Dimethylaminobenzo[m]−3-azapicene* (compound **XIII**). Olefin (compound **XII**) (0.25 g, 6.68E-4 mol) was dissolved in distilled THF (200 ml) in a flask equipped with a reflux condenser under argon and irradiated by an Hg high-pressure lamp for 40 h. The 24 h suggested in the original publication for a related compound (Fromherz et al., 2008) left unreacted 11-*N*,*N*-dimethylaminobenzo[m]−3-azapicene (compound **XIII**), as was apparent by reaction probes. At the end of the reaction, a dark precipitate developed on the walls that was not soluble in H_2_O, chloroform, or methanol, and very sparingly soluble in DMSO. After being washed with all of these solvents, the expected M^+^ peak of 373 for compound **XIII** could be detected but no further purification was conducted. Eighty milligrams of solid was obtained (32%) that was used in the next step.

**Figure 2.**
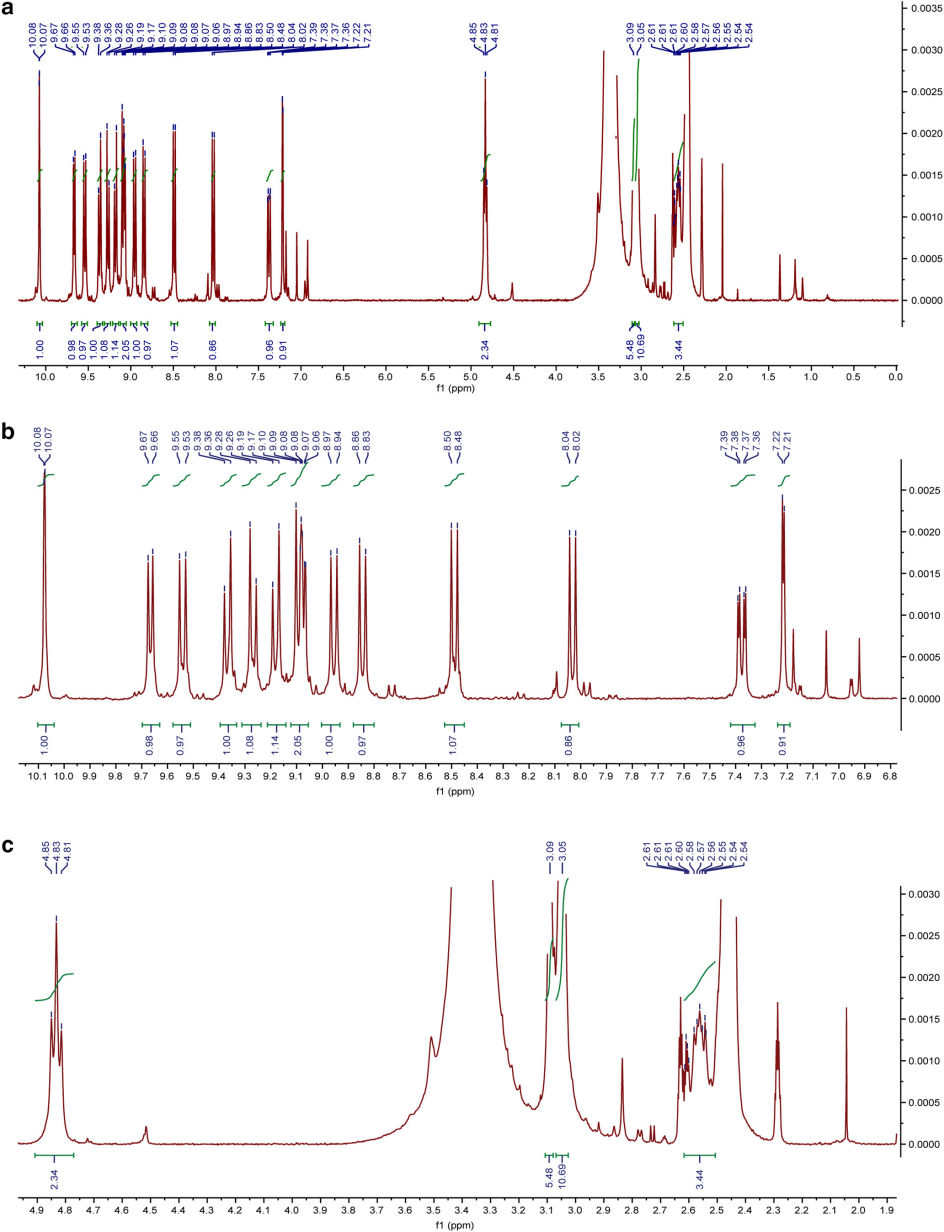
^1^HNMR of di1-ANNINE-6plus in DMSO-d_6_. ***a***, Full spectrum. ***b***, Aromatic expansion. ***c***, Aliphatic expansion.

**Figure 3.**
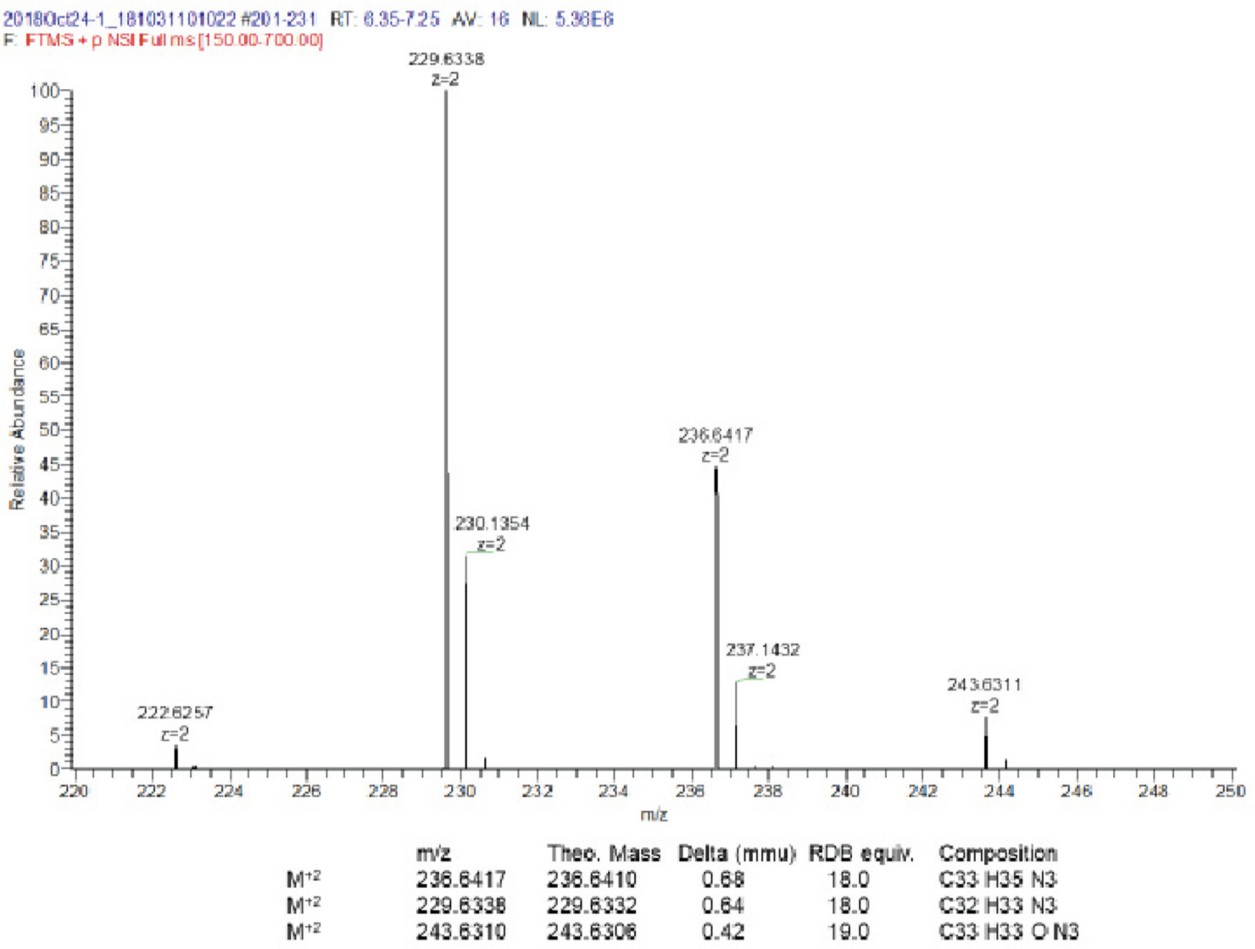
HRMS of di1-ANNINE-6plus. Expansion of relevant region.

*di1-ANNINE-6plus.* Eighty milligrams of compound **XIII** (2.15E-4 mol) was mixed with a 5× molar excess (0.28 g, 1.07E-3 mol) of (3-bromopropyl)-trimethyl-ammonium bromide (compound **XIV**) were mixed together in 3 ml of DMF and heated for 12 h at 120°C according to Fromherz et al. (2008). Subsequently a red precipitate fell out of the DMF solvent. The solvent was decanted, and the precipitate was washed with ∼30 ml of water. Since di1-ANNINE-6plus [compound **XV**; molecular weight (MW), 633.47] proved to be very sparingly soluble in water, it was possible to separate the well soluble excess compound **XIV**. 55 milligrams were obtained that could be further purified by HPLC (methanol/water mixtures) overnight to give 30 mg of >90% pure (25%) of compound **XV** that proved to be sparingly soluble in DMSO and DMSO/water mixtures; C_33_H_35_N_3_^2+^: MW calculated, 236.6410; high-resolution mass spectra (HRMS): 236.6417; ^1^H NMR (400 MHz, DMSO-*d*_6_): δ 10.08 (d, *J *=* *1.5 Hz, 1H), 9.67 (d, *J *=* *7.1 Hz, 1H), 9.54 (d, *J *=* *9.5 Hz, 1H), 9.37 (d, *J *=* *9.6 Hz, 1H), 9.27 (d, *J *=* *9.5 Hz, 1H), 9.18 (d, *J *=* *9.6 Hz, 1H), 9.12-9.05 (m, 2H), 8.96 (d, *J *=* *9.5 Hz, 1H), 8.84 (d, *J *=* *9.4 Hz, 1H), 8.49 (d, *J *=* *9.3 Hz, 1H), 8.03 (d, *J *=* *9.3 Hz, 1H), 7.38 (dd, *J *=* *9.3, 2.7 Hz, 1H), 7.22 (d, *J *=* *2.7 Hz, 1H), 4.83 (t, *J *=* *7.1 Hz, 2H), 3.09 (s, 6H), 3.05 (s, 9H), 2.62–2.51 (m, 4H) (Fig. 2). HRMS (Fig. 3).

#### Two-dimensional fluorescence spectrum of di1-ANNINE-6plus

**Figure 4.**
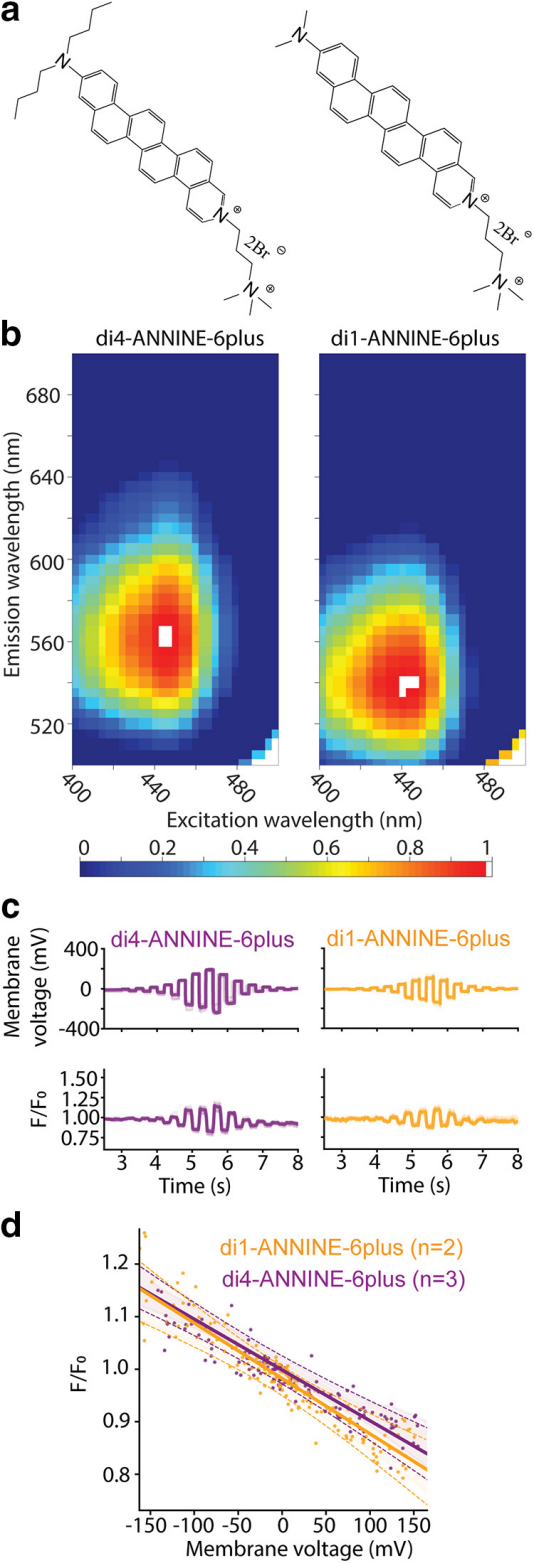
Structure, fluorescence spectrum, and sensitivity of di1-ANNINE-6plus. ***a***, Structure of di4-ANNINE-6plus (previously published under the name ANNINE-6plus) and di1-ANNINE-6plus. ***b***, 2D fluorescence spectra of both dyes measured in cortical tissue, normalized but otherwise not corrected. ***c***, HEK293 cells in a single confluent layer were simultaneously current injected with a patch pipette and voltage imaged (488 nm excitation, 561 nm long-pass filter). Recordings were made with di1-ANNINE-6plus and di4-ANNINE-6plus (orange and purple traces, respectively). ***d***, Fluorescence changes over membrane voltage change per voltage step (*n* indicates the number of cells). A linear regression was calculated for each dye (solid line), as well as the 95% confidence interval for each regression line (dotted lines), supporting that both dyes show the same sensitivity as expected from the same chromophores.

To determine the fluorescence characteristics of di1-ANNINE-6plus (Fig. 4*a*), we measured the two-dimensional fluorescence spectrum in brain tissue and compared it with the fluorescence of di4-ANNINE-6plus (Hinner and Hübener Sensitive Farbstoffe GbR; Fig. 4*a*; Fromherz et al., 2008). We homogenized freshly removed cortical brain tissue of a 2-month-old male mouse and labeled it with di1-ANNINE-6plus or di4-ANNINE-6plus (both, stock solution 2.3 mm in DMSO, then diluted to 20 μm in 0.9% NaCl saline). We used a TIDAS S 700 UV/NIR 2098 Diode Array Spectrometer coupled to a TIDAS S Monochromator-VIS with 75 W Xenon light source (J&M Analytik AG). Excitation wavelength was varied from 390 to 500 nm, in 5 nm steps, while the emitted light was collected with a diode array from 200 to 980 nm with 5 nm resolution. Data were exported to MATLAB (MathWorks) to create 2D spectra graphs, normalized to the global peak intensity (Fig. 4*b*).

#### Sensitivity testing of di1-ANNINE-6plus in HEK 293

After the addition of di1-ANNINE-6plus (0.75 ml of 0.5 mm in saline) or di4-ANNINE-6plus (0.5 ml of 2 mm in saline) to the culture dish with 2 ml of DMEM culture medium (Wako), HEK293 cells were placed under a bright-field microscope (BXW51, Olympus) magnified 60× (LumPlanFL N 60×/1.00 W, Olympus) and maintained for 20–30 min at room temperature for labeling. Cells were selected depending on their good adherence to the Petri dish, being the right level of confluent (attached to other cells, but also with enough space to be optically well defined), and patch-clamped in their original DMEM culture medium, with a borosilicate patch-clamp electrode (R = 10 MΩ) containing 120 mm potassium gluconate, 7 mm KCl, 10 mm HEPES, 0.1 mm EGTA, and 2 mm ATP disodium salt hydrate, adjusted to pH 7.25 and 300 mOsm. For membrane potential acquisition, we recorded individual HEK cells (*n* = 5) in whole-cell current-clamp configuration with a Double IPA integrated patch amplifier assisted by SutterPatch software (Sutter Instruments). Each cell was stimulated with 12 × 200 ms positive and negative square current pulses, increasing from ±0.1 to ±4 nA. For voltage imaging, we illuminated the preparation with a 488 nm wavelength laser source (3 W custom 488 nm laser; Genesis MX-488, Coherent) through a patterned illumination system with digital micromirrors (Mosaic3, Andor Technologies). Emitted fluorescence was high-pass filtered at 561 nm (RazorEdge ultrasteep long pass edge filter, Semrock), and signal was recorded with a CMOS camera (256 × 256 pixels, 40 fps; MiCAM03, BrainVision). Analyses (Fig. 4*c*,*d*) were performed with home-made python routines (WinPython 3.3.5, Python Software Foundation) based on custom scripts. Statistical analyses (see Fig. 4*d*) were performed using the SciPy plugin.

#### Animal experiments

All animal experiments were performed in accordance with guidelines approved by the Institutional Animal Care and Use Committee in an Association for Assessment and Accreditation of Laboratory Animal Care (AAALAC International)-accredited facility.

#### Surgery and dye application

The surgeries conducted here were an alteration of the standard chronic cranial window surgeries (Holtmaat et al., 2009; Roome and Kuhn, 2014). Male C57/BL6 mice (*n* = 7; age, 31–180 d) were anesthetized with isoflurane at 2% in air and head fixed in a stereotactic frame. During the surgery, isoflurane was reduced to 0.5–1.0%. First, the eyes of the mouse were protected from debris and drying with an eye ointment. Then hair on the scalp was removed with an electric shaver, and hair removal cream. Carprofen (5 μg/g body weight) and buprenorphine (0.1 μg/g body weight) were injected subcutaneously, and dexamethasone (2 μg/g body weight) was injected intramuscularly, into the hindlimb. The scalp was then cleaned with iodine solution and numbed with lidocaine gel before being opened with a scalpel. A pair of surgical scissors was then used to remove a triangular flap of skin, exposing the skull from the anterior of bregma, to posterior of λ, going slightly right of the midline, and exposing the entire parietal bone on the left. The bone was then dried and cleaned with compressed air, and scrubbed with sterile cotton swabs soaked in lidocaine solution. The craniotomy was then marked out above barrel cortex (center anteroposterior, −1.5 mm; lateral, 3 mm, relative to bregma; radius, 2 mm), and bone surrounding it was carefully thinned and smoothed. A channel around the remaining circular bone patch was made, allowing it to float freely from the rest of the skull. Care was taken not to damage the dura mater. Following Roome and Kuhn (2014), a wooden toothpick was glued vertically onto this free-floating bone, using superglue (Fig. 3*a*). If electroencephalogram (EEG) wires were needed, they would be implanted at this point. For electroencephalogram wires, two silver leads (0.404 mm thick, 2–3 cm long) would be prepared by hammering the last 5 mm flat. Two small holes would then be drilled, one lateral to the craniotomy, and the other on the opposite hemisphere, symmetrically opposed to the craniotomy, mostly through the skull, where the wire should be placed. Then the hole would be finished with a small cut from a 26 gauge beveled needle, leaving the dura undamaged. The wire was placed into the hole, flat on the dura (3–5 mm) and then superglued in place, ensuring the wire lead also lay flat on the skull. After both wires were glued in place, preparation to open the craniotomy could begin.

**Figure 5.**
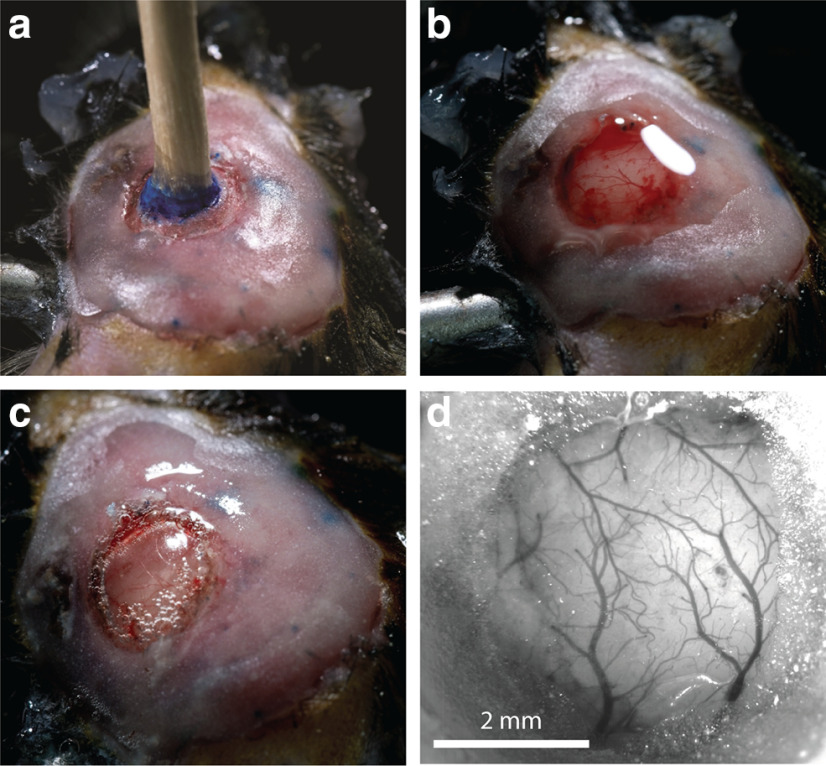
Cranial window surgery. ***a***, After drilling the channel through most of the bone around the circular bone patch, a wooden toothpick was glued to the circular bone patch. Dental cement (pink) covers the bone beyond the window area. The blue ink marked the position of the craniotomy. ***b***, Using the toothpick as a handle, the bone patch was gently removed, and saline was added to wash away any blood. ***c***, Hydrogen peroxide was added to the dura so that a bubble forms. Gel foam, forming a well on the craniotomy, was removed for clarity. ***d***, After dye injection, the bubble below the dura was deflated during the mounting of the window glass. The cranial window was clear and ready for imaging.

Dental acrylic (SuperBond) was next applied around the edges of the craniotomy, forming a well. Care has to be taken that no dental acrylic leaks into the channel between the skull and the free bone. This acrylic was built up to create a flat surface that would later be used to mount the head plate. If electrodes were present, some acrylic at this point was also applied to the base of the electrode leads, helping to cement them in place and isolate them. Buildup of acrylic (Fig. 5*a*) was limited to <1 mm, however, so that the eventual mounting of the head plate would not prevent the microscope objective from reaching its required depth.

After the acrylic dried, the floating bone patch could be removed, carefully cracking all the edges of the channel by rocking the toothpick slowly back and forward, before slowly peeling the bone away, using the toothpick as a handle. If done gently, the dura will remain intact, and there should be little to no bleeding (Fig. 5*b*). In the next, most critical step, a bubble will be formed under the dura. This technique is modified from that described before, for removing the dura mater in a mouse surgery (Sheroziya and Timofeev, 2014). To do this, gel foam that had been soaking in a mix of saline and carprofen solution (ratio, 1:10) was gently packed against the edges of the craniotomy, so that the hard bone would not damage the dura during this extended opening of the skull. Next, dilute hydrogen peroxide (1.4% H_2_O_2_ in saline was dripped onto the exposed dura, using an eyedropper. Drips were added until the dura had puffed up and separated from the brain below. This takes typically 5–10 s and requires 1–10 ml of H_2_O_2_ solution. Saline was then used to wash away the peroxide. The dura bubble was continuously kept wet with saline (Fig. 5*c*).

Next, a quartz micropipette beveled to a 20 μm opening was tip filled with 200–1000 nl of di1-ANNINE-6plus solution using suction. The stereotaxic frame was used to position this pipette above the dura bubble and then slowly enter it, being careful not to touch the brain itself. Once the tip was inside the bubble, the contents of the pipette were pressure ejected, but making sure not to push air into the dura bubble. After this, the pipette was carefully removed. The pipette could then be reloaded with dye and deliver several microliters of dye inside the dura bubble, typically 3–8 μl. Five to 10 min was given to let the dye diffuse, before putting a 5-mm-diameter glass window (170 μm thick) onto the dura bubble and slowly pressing it down, thereby deflating the bubble. The window was then glued in place to the bone edges of the craniotomy, using thin superglue, applied with a fine-tipped transfer pipette. Dental acrylic (SuperBond) was applied to cover all remaining bone and to cement a head plate onto the skull. Finally, the mouse was transferred without waking from anesthesia to the microscope for functional imaging.

#### Testing for peroxide effect on cortex

A mouse expressing GCaMP6f in cortical cells underwent a bubble surgery to test the effects of peroxide application to the dura on cortical cells. This was done with an initial surgery creating a small craniotomy above barrel cortex, where a GCaMP6f-encoding viral vector was injected into the cortex (AAV1.Syn.Flex.GCaMP6f.WPRE.SV40, 1.4E13 genome copies/ml; and AAV1.hSyn.CRE.WPRE.hGH, 5.6E10 genome copies/ml, both AddGene; ratio, 1:1; 70 nl injected 400 μm below dura mater). The craniotomy was then sealed with self-curing silicone gel (Kwik-Cast, World Precision Instruments), the scalp was glued back together with superglue, and the mouse was returned to its home cage to recover. After 2 weeks, the mouse underwent a bubble surgery, which was performed over the previously made craniotomy. This bubble surgery was performed without voltage dye. After the peroxide was washed away and the dura allowed to deflate, a window and head plate were cemented over the craniotomy and the mouse was transferred directly to a two-photon imaging setup, where dendrites of cortical neurons were inspected for breaks and calcium activity.

#### Testing for peroxide effect on immune response

To determine the level of immune response caused by the bubble surgery compared with standard chronic window surgeries, immunofluorescence staining was performed on brain slices, taken from animals after either bubble or chronic window surgeries. Two time points were compared: 3 h postsurgery, aligning with the time acute imaging would be performed for the bubble surgeries described in this article; and 1 week postsurgery. In the 1 week postsurgery case for bubble surgery, the bubble was deflated, and a chronic cranial window was mounted. In all cases, mice were killed and perfused with saline, and then periodate-lysine-paraformaldehyde (PLP; 4% paraformaldehyde in PBS, with 0.1 m L-lysine, and 0.01 m sodium meta-periodate). Following perfusion, the brains were kept in PLP for 2 d before being switched to a 20% sucrose solution in PBS for a day. The sections of interest were then cut out and frozen in optimal cutting temperature compound (O.C.T. compound, Tissue-Tek), at −80 °C, so they could be sliced using a cryostat microtome (40 μm sections, −20°C; CM3050-S, Leica). Alternating slices were selected for glial fibrillary acidic protein (GFAP; labeled with rabbit anti-GFAP, ab7260, Abcam) or ionized calcium-binding adapter molecule 1 (IBA1; labeled with rabbit anti-IBA1, ab178846, Abcam) staining. Staining followed the same protocol for each antibody. Slices were kept in free-floating wells, with 500 μl of PBS, and then washed four times in PBS, with agitation and fresh PBS. Blocking was conducted by removing the PBS and washing the slices in 200 μl of blocking solution [10% goat serum albumin (GSA), 5% bovine serum albumin (BSA), 0.3% Triton, in PBS], under agitation, for 3 h at room temperature. The blocking solution was then replaced with 150 μl of primary antibody solution (1:2000 GFAP antibody in blocking solution or 1:500 IBA1 antibody in blocking solution), and left on agitation, at 4°, overnight. The following day, these were washed three more times in PBS at room temperature, before the secondary antibody exposure [200 μl of 1:200 goat anti-rabbit IgG (H+L) Alexa Fluor 488, ab150077 (Abcam) in PBS, with 5% GSA, 2% BSA, and 0.3% Triton], in the dark, with agitation, for 4 h. This was then washed off with three washes of PBS and agitation. Slices were then mounted on glass slides and imaged with a mosaic scanning fluorescence microscope (ECLIPSE N*i* with DS-Ri2 color camera and NiS software, all from Nikon; laser excitation, 488 nm; fluorescent light collected through 510–560 nm emission filter).

#### Imaging setup

Imaging was performed with a custom wide-field and two-photon microscope (MOM, Sutter Instruments). A 2.5× air objective (A-Plan 2.5×/0.006, Zeiss) was used for wide-field imaging to inspect the window (Fig. 5*d*), and a 25× water-immersion objective (XLPlan N 25×/1.05 W MP, Olympus) for two-photon imaging. The tuning collar for the 25× objective was purposefully defocused, so the point spread function would be extended to 5 μm in *z*-direction (Roome and Kuhn, 2018, their Supplementary Fig. 10). di1-ANNINE-6plus was excited with 1020 nm from a pulsed Ti:sapphire laser (Chameleon Vision II, Coherent) and collected with a GaAsP photomultiplier tube (Hamamatsu) without emission filters. For functional scans, 250 μm line scans (512 pixels) were collected at 2 kHz. The typically used laser power was ∼60 mW, measured at the objective front lens.

Functional scans were taken after di1-ANNINE-6plus diffused through L1, ∼30–45 min postsurgery. Anesthetized recordings were made first (isoflurane, 0.5–1% in O_2_; body temperature, 37°C). For awake recordings, the heating pad and temperature probe were removed, the isoflurane was turned off, and the mouse was allowed 15–20 min to wake up. Once the heating pad was removed, the treadmill wheel that the mouse was sitting on would become free, so the awake mouse could run if it chose to.

#### Data analysis

All functional image data were imported into MATLAB (MathWorks) and processed using custom software. All data were collected as line scans at 2 kHz, for 11 s (512 × 22,000 pixels). Line-scan files were processed separately, before averaging. In every line-scan file, time series of four pixel blocks were averaged (resulting in 128 time traces), a frequency power spectrum calculated (128 spectra; frequency range, 0.09 Hz to 1 kHz), and then averaged. Spectra were then averaged based on cortical depth (imaged at 25, 50, 75, and 100 μm below dura mater) and behavioral state (awake or lightly anesthetized). These averaged spectra were normalized by calculating $S(f)=\frac{s(f)-s_{0}}{s_{0}}$, where $s_{0}$ is the average power in the frequency range between 40 and 50 Hz, where we noticed no peaks or activity in individual spectra. Some artifacts were detected, indicated by sharp frequency peaks. All artifacts were >50 Hz, where we could not detect any noticeable oscillation peaks, so the spectra were trimmed to 50 Hz. No filters were applied to the raw voltage data or frequency spectra. Artifacts due to breathing (typical frequency, ∼2.5 Hz) and heartbeat (typical frequency, ∼8–10 Hz) were not observed.

## Results

### Two-dimensional fluorescence spectrum of di1-ANNINE-6plus

di4-ANNINE-6 (Hübener et al., 2003) shows many useful features for voltage imaging; however, it is not soluble in water or saline and it diffuses very slowly into tissue (B Kuhn; unpublished observation). Previously, we improved the water solubility by replacing the negative charge of the headgroup with a positive charge leaving the chromophore unchanged, resulting in di4-ANNINE-6plus (Fig. 4*a*, left). As the chromophore remained unchanged, the voltage sensitivity did not change (Fromherz et al., 2008). To increase diffusion in tissue, here we shortened the carbohydrate tail from di-butyl to di-methyl, again without changing the chromophore, resulting in di1-ANNINE-6plus (Fig. 4*a*, right). Despite the shorter carbohydrate tail, di1-ANNINE-6plus is less water soluble than di4-ANNINE-6plus (<0.1 mm and ∼4 mm, respectively). This is most probably due to stacking of the chromophore ring system. The advantage of the di1-ANNINE-6plus lies not in the better solubility in water but in the faster diffusion in tissue (see below).

To compare di4-ANNINE-6plus and di1-ANNINE-6plus, we measured two-dimensional fluorescence spectra. As voltage-sensitive dyes, including ANNINE dyes, show strong solvatochromic effects, we measured the spectra under conditions relevant for functional imaging (i.e., in cortical brain tissue; Fig. 4*b*). The excitation peaks of both dyes occur at 435 nm, while the emission peak is slightly shifted from 560 nm for di4-ANNINE-6plus to 540 nm for di1-ANNINE-6plus. Importantly, the red spectral edge of absorption of di1-ANNINE-6plus is the same as for di4-ANNINE-6 (Kuhn et al., 2004) and di4-ANNINE-6plus (Fromherz et al., 2008). The red spectral edge of absorption is the wavelength range where voltage-sensitive dyes should be excited for highest sensitivity and lowest phototoxicity and bleaching (Kuhn and Roome, 2019).

### Sensitivity of di1-ANNINE-6plus

As expected, comparing di1-ANNINE-6plus to di4-ANNINE-6plus, here under one-photon excitation, found no significant difference in voltage sensitivity (Student’s *t* test, *p* = 0.24; Kolmogorov–Smirnov test, *p* = 0.45; Fig. 4*c*,*d*). Sensitivity was tested over −150 to 150 mV and was linear throughout that range. The slope of the regression is the voltage sensitivity of the dye (−9.7 ± 2.9%/100 mV and −10.3 ± 3.9%/100 mV, respectively. Both regression lines overlap their confidence intervals, indicating no significant difference between them. Additionally, a Student’s *t* test found no significant difference between the sensitivity of the two dyes, and a Kolmogorov–Smirnov test found *p* = 0.45, so above the threshold for statistical similarity (*p* > 0.41). As the cell cultures were confluent, the determined sensitivity is lower than previously reported for ANNINE-6 excited at 488 nm [−28%/100 mV (Kuhn et al., 2004)] due to fluorescence background by neighboring cells.

### Immune response of bubble surgery

**Figure 6.**
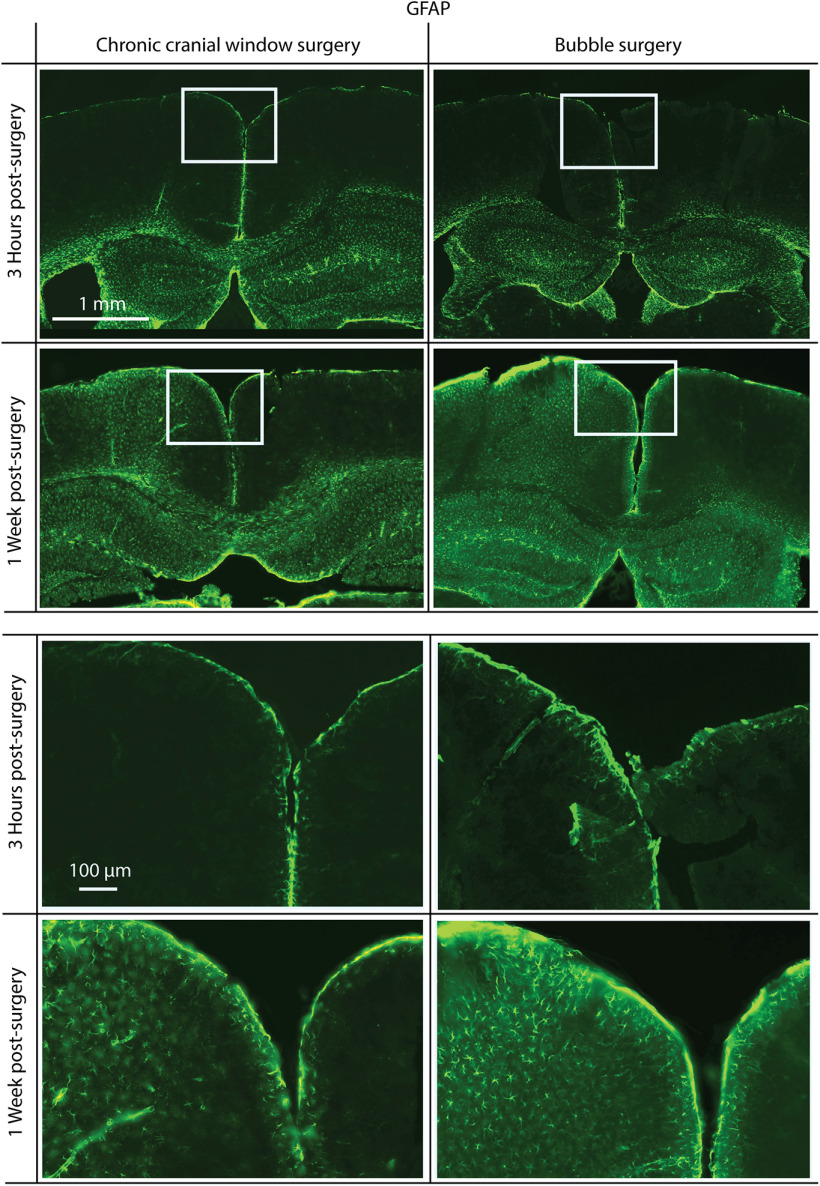
Immunofluorescence staining for GFAP, a reactive astrocyte-specific marker. Mice were killed 3 h (top) or 1 week (bottom) postsurgery. Surgeries were either a standard chronic cranial window surgery (left) or a bubble surgery (right). There is no noticeable increase in GFAP 3 h following surgery, but in both chronic cranial window and bubble surgery preparations there is a similar increase 1 week postsurgery. Surgery was performed on the left hemisphere in each coronal slice shown. White rectangles indicate the positions of magnified areas (lower half).

**Figure 7.**
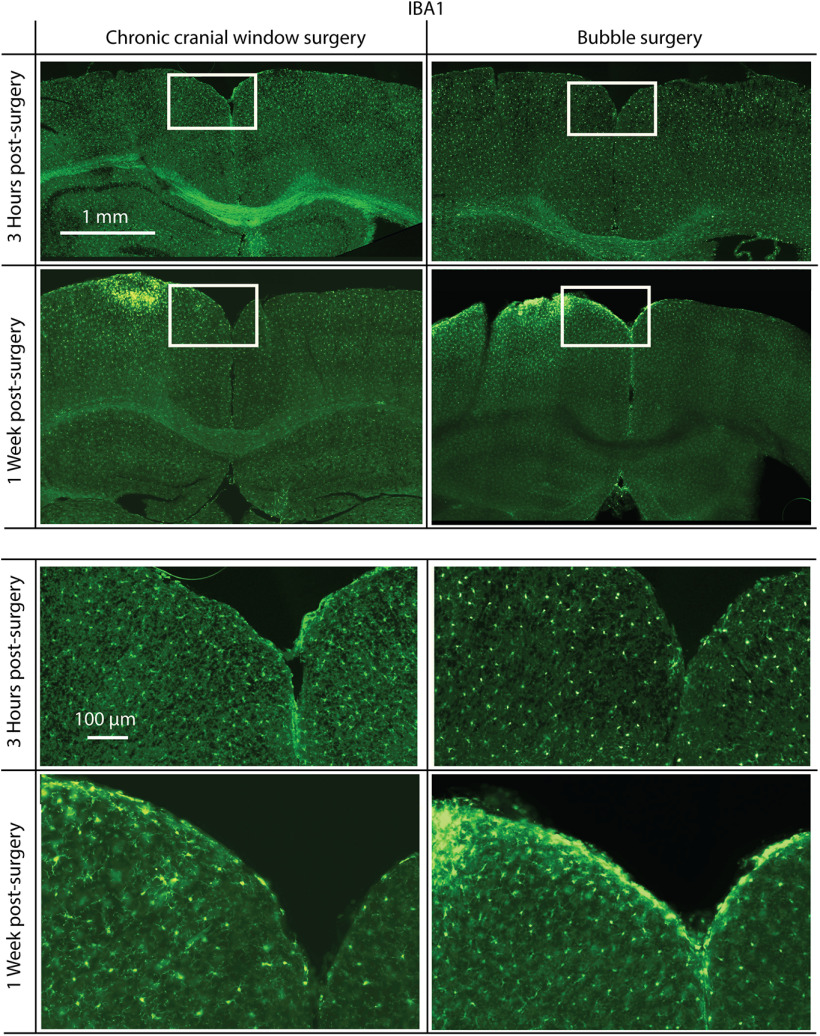
Immunofluorescence staining for IBA1, a microglia activation-specific marker. Mice were killed 3 h (top) or 1 week (bottom) postsurgery. Surgeries were either a standard chronic cranial window surgery (left) or a bubble surgery followed by the mounting of a chronic cranial window (right). There is no noticeable increase in IBA1 3 h following surgery, but in both chronic cranial window surgery and bubble surgery preparations there is a similar increase in IBA1 1 week postsurgery. Surgery was done on the left hemisphere in each coronal slice shown. White rectangles indicate the positions of magnified areas (lower half).

To test whether the bubble surgery, and specifically the peroxide, causes an immune response, we antibody labeled brain slices postsurgery and postrecovery with markers for increased glia activity, GFAP and IBA1. In both bubble surgery and standard chronic cranial window surgery, 3 h following surgery, there was no noticeable difference between the hemisphere, which underwent surgery, and the undisturbed hemisphere in both preparations (two mice each), in either GFAP (Fig. 6) or IBA1 (Fig. 7) staining. After 1 week, there was an increase in GFAP and IBA1 for both surgical procedures compared with the undisturbed hemisphere. However, we found no noticeable difference in GFAP and IBA1 increase between bubble surgery and standard chronic cranial window surgery, confirming the viability of the bubble surgery.

### Labeling of cortical L1

**Figure 8.**
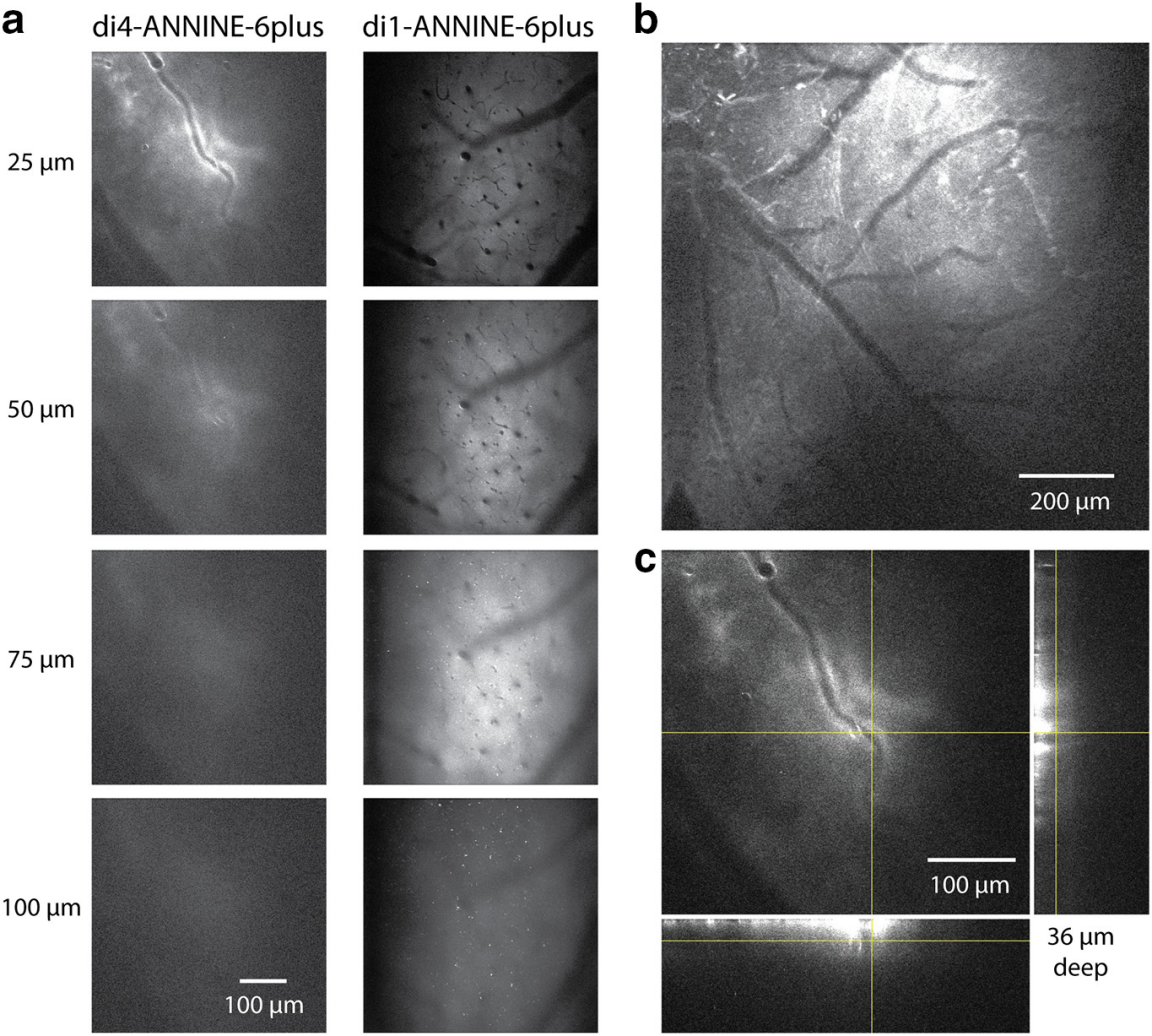
Comparison of labeling with di4-ANNINE-6plus and di1-ANNINE-6plus after bubble surgery. ***a***, Images taken from an image stack of di4-ANNINE-6plus (left) and di1-ANNINE-6plus (right) staining in barrel cortex *in vivo*. Following the same cortical surface application protocol (i.e., filling the bubble with staining solution), di1-ANNINE-6plus diffuses deeper into cortex. ***b***, Two-photon image of di1-ANNINE-6plus spread over cortical L1, following bubble surgery. Dye is spread >1.2 mm laterally across the cortex. ***c***, Image stack of di4-ANNINE-6plus applied in a bubble surgery. The deepest point at which dye reliably reached is ∼35–40 μm below the dura. Orthogonal projections of the image stack at the crosshairs show the extent of the dye penetration through L1, which is notably less than that of di1-ANNINE-6plus.

To compare the tissue-labeling properties of di4-ANNINE-6plus and di1-ANNINE-6plus, we used both with the above-described bubble surgery under the same conditions (Fig. 8*a*). di1-ANNINE-6plus yielded better labeling, as it penetrated deeper into cortex (Fig. 8*a*) and spreads out widely (Fig. 8*b*), while di4-ANNINE-6plus sticks to the surface (Fig. 8*c*). This increased penetration into tissue occurs most probably due to weaker membrane binding of di1-ANNINE-6plus, which in turn increases diffusion.

**Figure 9.**
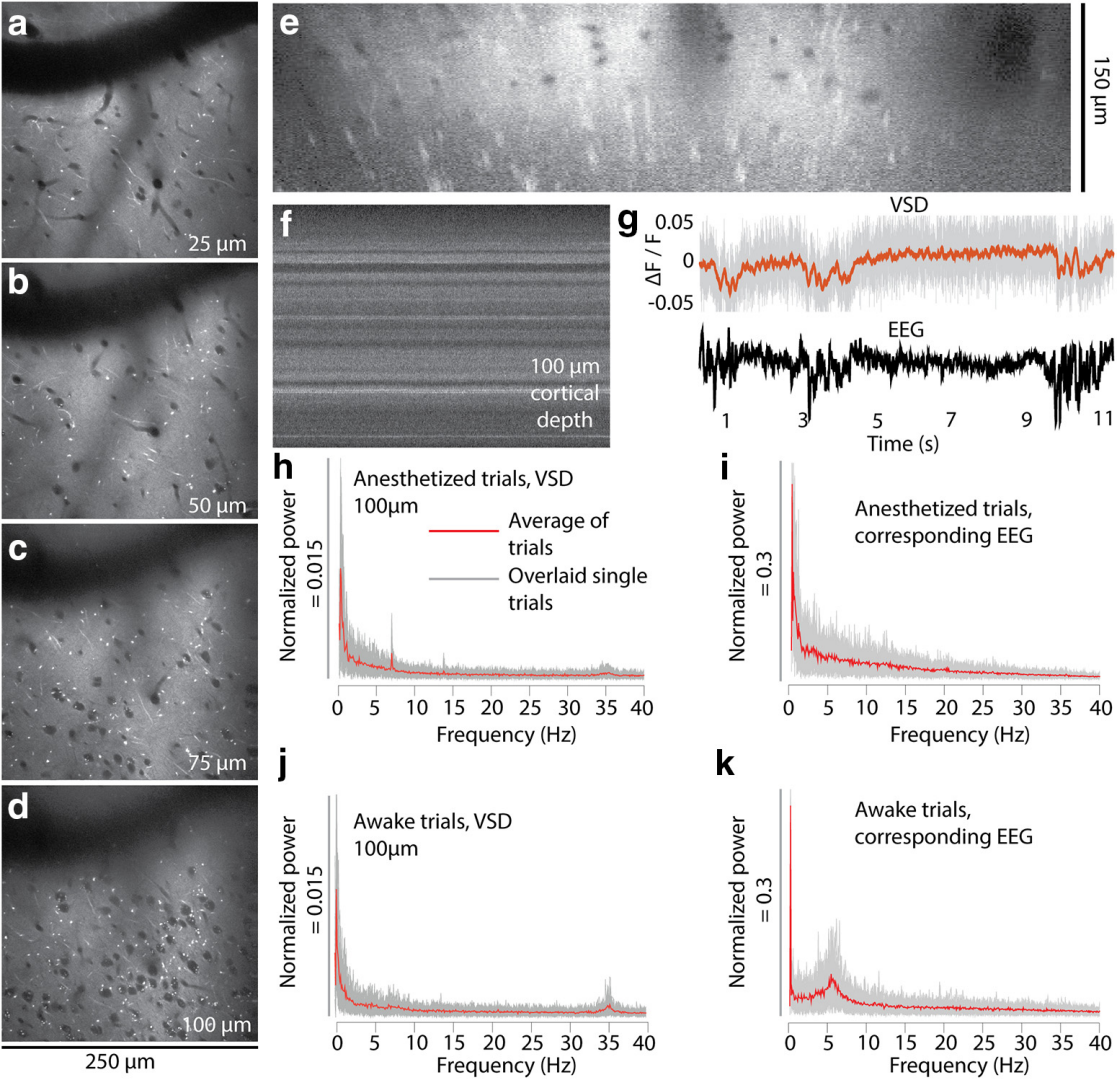
*In vivo* two-photon imaging of cortical L1 and 2 after labeling with di1-ANNINE-6plus. ***a–e***, Labeling with di1-ANNINE-6plus *in vivo* in different depths (***a–d***) and the corresponding *x*–*z* reconstruction (***e***). Bright spots indicate lipid-rich structures, most likely myelinated axons. ***f***, ***g***, Line-scan recordings (2 kHz recording; dwell time, 1 μs/pixel; ***f***) and example of paired VSD and EEG recordings (***g***; 11 s anesthetized trace, 75 μm below dura; gray, raw VSD data; red, filtered recording; low-pass filtered at 30 Hz, eighth order; black, EEG) under isoflurane anesthesia showing spindle activity. ***h–k***, Frequency spectra of membrane voltage and corresponding EEG recordings calculated from 30 recordings (11 s each; gray background, overlay of single trials; red line, average) in an awake and anesthetized mouse.

di1-ANNINE-6plus reliably penetrates the upper 150 μm of cortex within 45–60 min (Fig. 9*a–e*). Because of the extracellular application of the dye, somata become visible as shadows. Bright processes, visible in the sections as bright spots, indicate most likely membrane-dense structures, like myelinated axons. The strong labeling lasts for ∼48 h.

### Testing for peroxide effect on cortex

**Figure 10.**
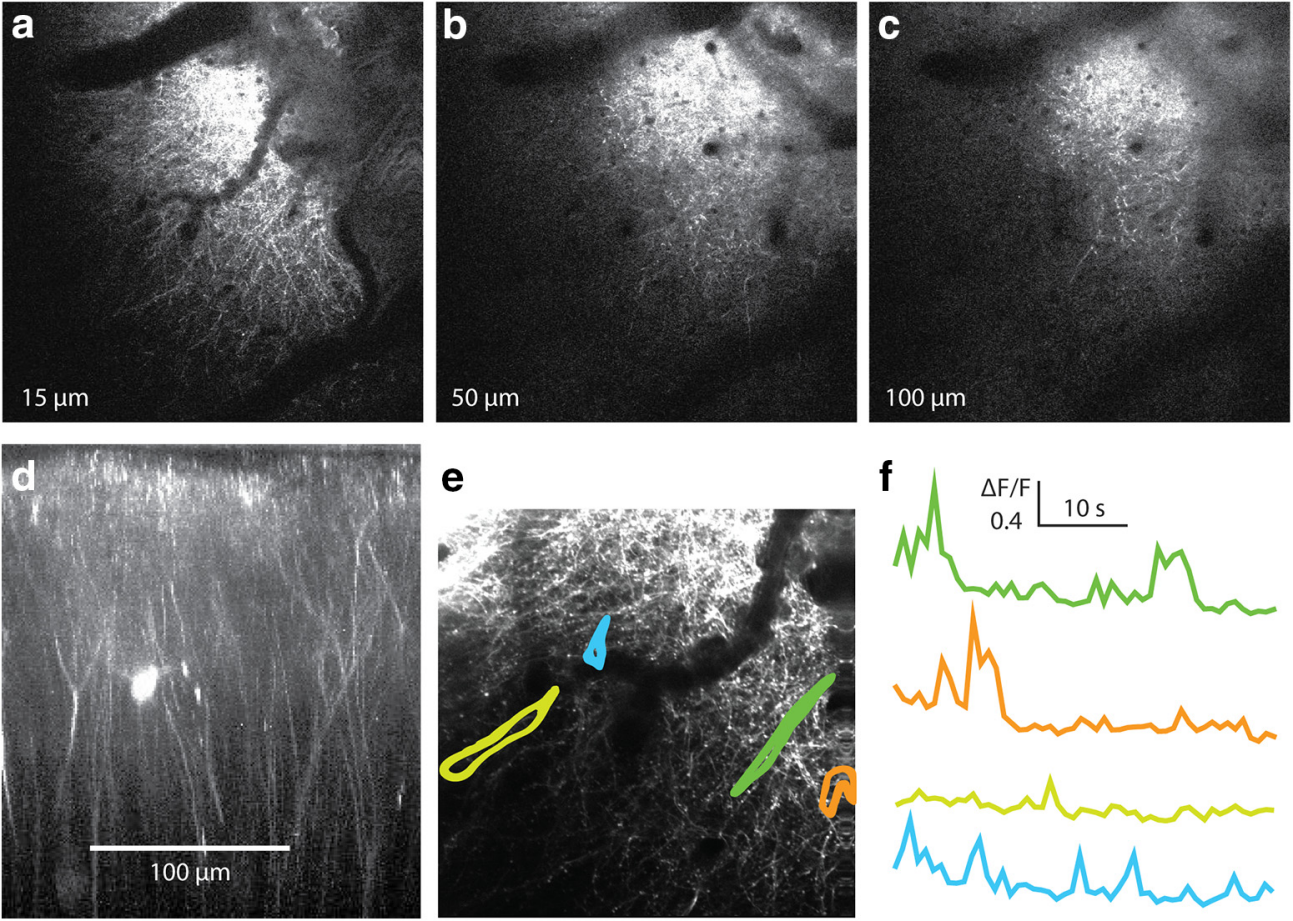
Two-photon calcium imaging of L1 fibers after bubble surgery. ***a–c***, Successive slices in an image stack with intact fibers throughout L1. ***d***, Reconstruction of the image stack. The reconstruction confirms that the bubble surgery does not affect L1. ***e***, A recording during wakefulness was taken in L1. ***f***, Individual regions of interest, indicated in ***e***, show typical dendritic calcium activity. Recordings were taken at 4 Hz. This supports the observation that bubble surgery and peroxide application do not disrupt neuronal processes or kill neurons in the upper cortical layers.

We tested whether the bubble surgery affects L1 by imaging the dendritic structure and calcium activity in L1 (Fig. 10). The dendritic structure appeared regular, and no broken dendrites, swellings, or other obvious damage were detected. Also, calcium signals were regular transients, and no unphysiological signals were observed.

### Cortical membrane voltage oscillations in L1

To analyze cortical membrane voltage oscillations, line scans were recorded avoiding large blood vessels or their shadows. After recording a line scan (Fig. 9*f*, example), it was spatially averaged (Fig. 9*g*, top). Under anesthesia, spindle activity recorded in the EEG (Fig. 9*g*, bottom) was visible with a low signal-to-noise ratio (<2) in the averaged line scan (Fig. 9*g*, top) but could be improved by temporal filtering. As expected, the depolarizing spindles result in a negative relative fluorescence change (Fig. 9*g*, top) and in corresponding multiphasic EEG signals (Fig. 9*g*, bottom).

**Figure 11.**
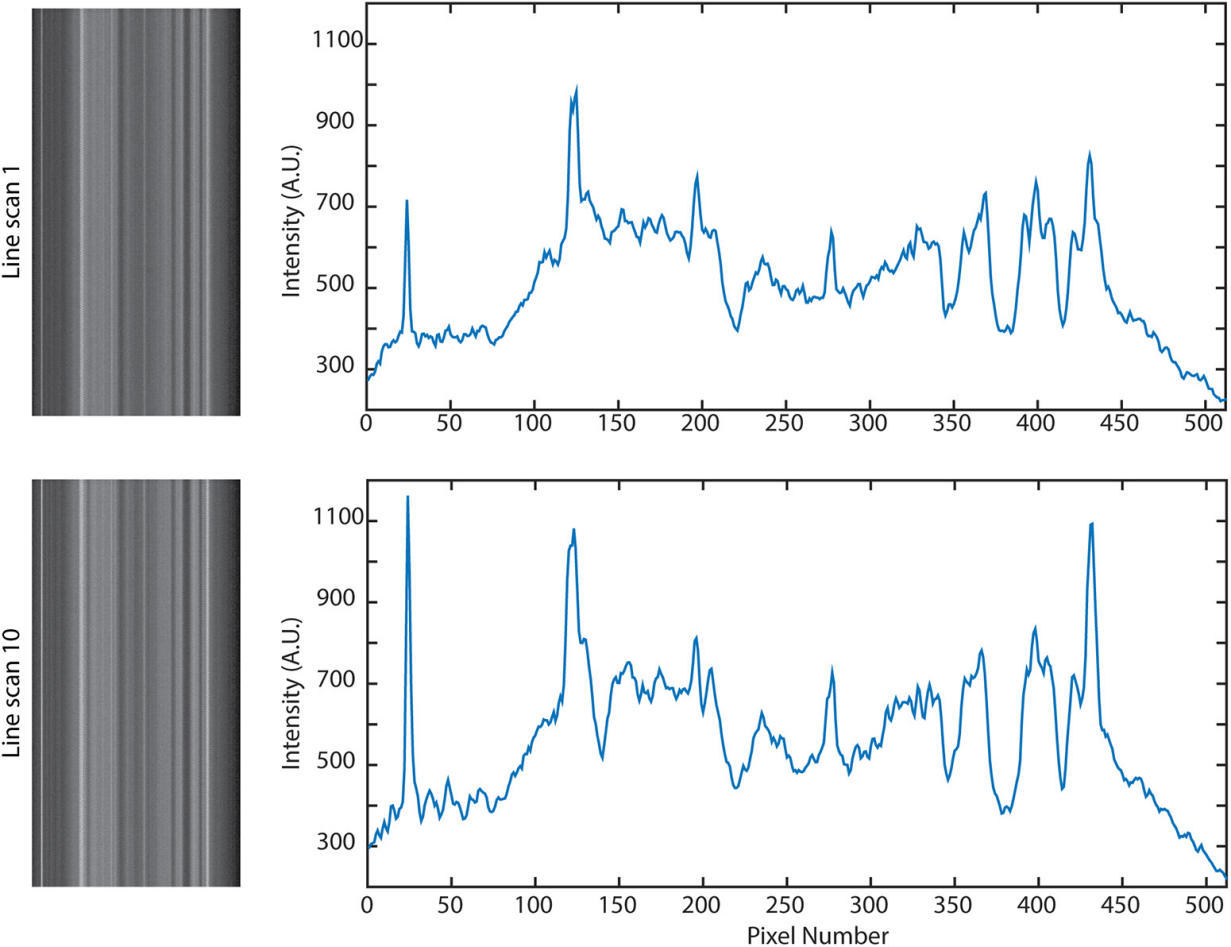
Consecutive line scans over the same area do not cause bleaching. Over 10 consecutive line scans (11 s each, during a 5 min period), the brightness did not decrease, although small changes have occurred due to movement.

Importantly, despite extended line scanning, bleaching was negligible, and the fine pattern of membrane changed only slightly (Fig. 11).

From the spatially averaged line scans recorded under light anesthesia or in the awake state, frequency spectra were calculated and averaged (Fig. 9*h*,*j*). The averaging of spectra reduces the relative noise with the square root of the number of averages. We recorded for 330 s (30 averages of 11 s recording) to reliably determine cortical membrane voltage oscillations in a frequency range from 0.5 to 40 Hz and the EEG, both under light isoflurane anesthesia (Fig. 9*h*,*i*) and in the awake state (Fig. 9*j*,*k*). Different frequency bands can be observed, especially at <10 Hz and ∼35 Hz. We find two prominent frequency bands. First, the spectrum of the anesthetized recording at <10 Hz is dominated by a narrow frequency band at ∼7 Hz, which is absent in the spectrum of the awake recording. This band is also not visible in the EEG spectrum. Second, a frequency band at ∼35 Hz, which is not visible in the EEG spectrum, has ∼50% more power amplitude in the spectrum of the awake recording (FWHM = 1.3 Hz) than the anesthetized recording (FWHM = 1.64 Hz). A strong oscillation band at ∼6 Hz in the EEG spectrum (Fig. 9*k*) has in this example no correlated membrane voltage oscillation in L1 (Fig. 9*j*).

**Figure 12.**
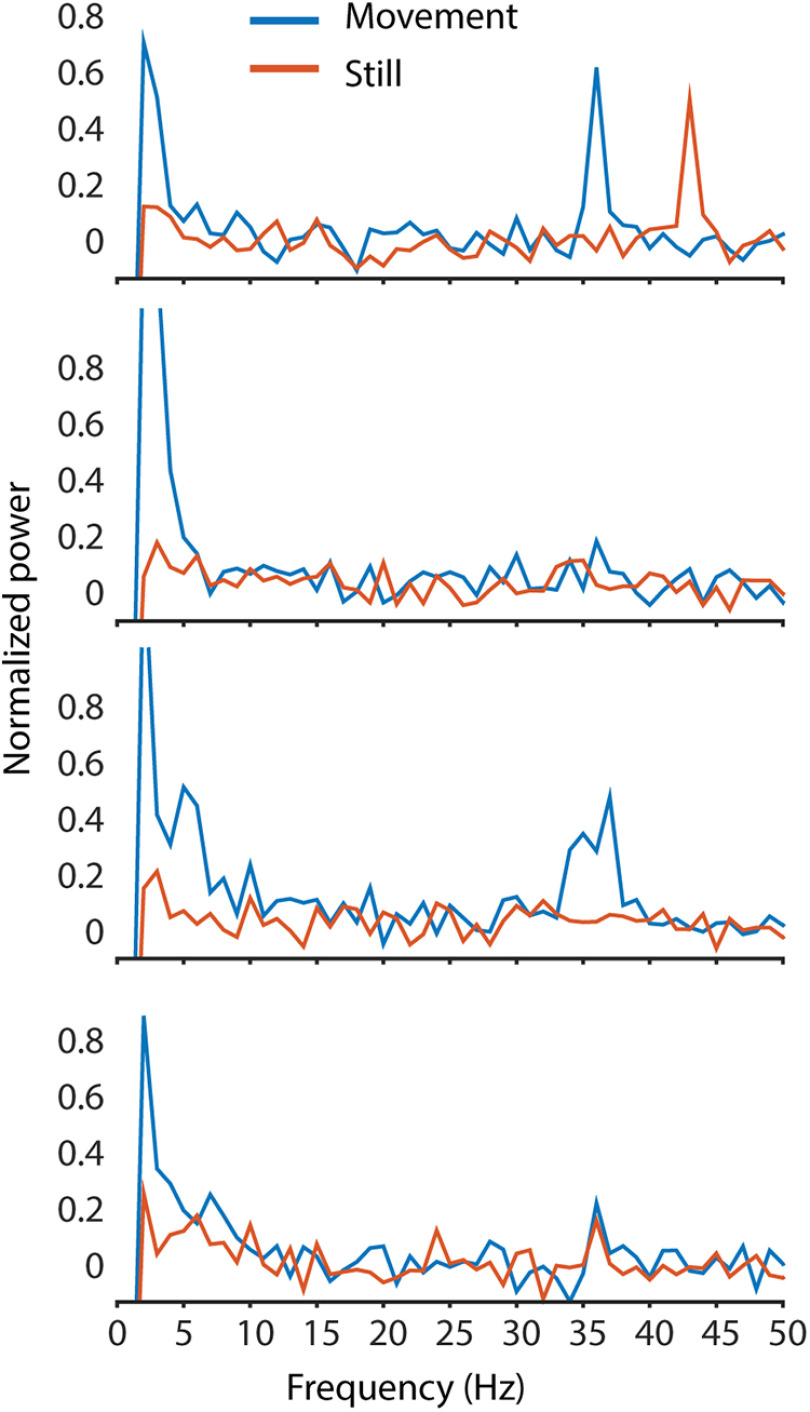
Example spectra from awake recordings with 1 s movement segments separated from 1 s still segments (based on rotation of the treadmill during the 1 s epoch). Movement alters oscillations; however, peaks can be found in both movement and still periods. The small segment size used reduces the frequency resolution of these spectra to 1 Hz, and the low-frequency cutoff to 1 Hz. The number of epochs averaged for each movement trace in each respective graph (top to bottom) are 69, 205, 194, and 161, 1 s each; the number of still epochs averaged are 261, 125, 136, and 169, 1 s each.

During some recordings, the movement of the mouse was recorded via a rotary encoder built into the treadmill the mouse was sitting on. The recordings from these trials were then split into 1 s segments, before being grouped in either movement or still periods, depending on whether any movement was detected in the rotary encoder during the second. This included running and small movement twitches, to ensure that no movement artifacts were left in the nonmovement segments. The frequency spectra of the 1 s segments were averaged, and the oscillations in each of these groups were compared (Fig. 12). All oscillation peaks were detected in both movement and still periods, although there may be some difference between the exact peak location and power. Because of the sampling rate limitations of the data acquisition, and the small period of time for each segment, further data analysis used the entire recording with movement and still periods.

**Figure 13.**
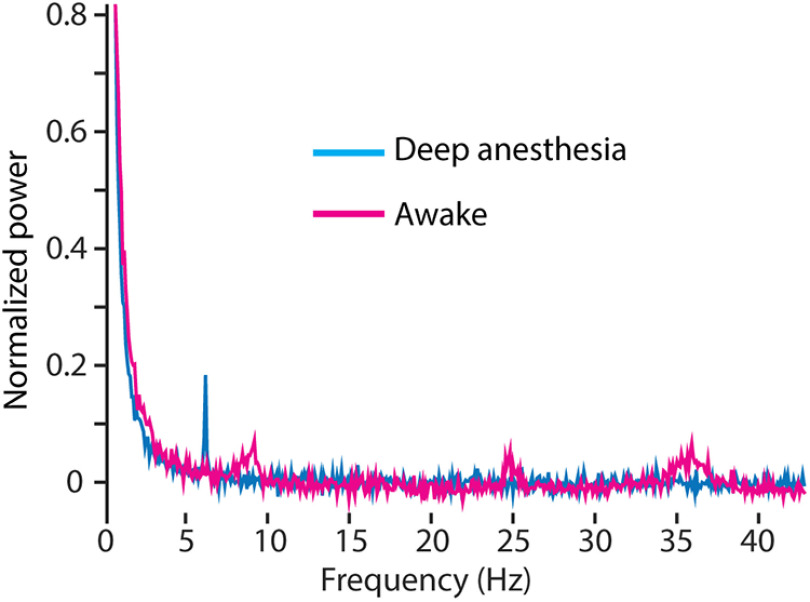
Gamma and other oscillations disappear under deep anesthesia. Spectrum recorded under awake conditions (magenta) was acquired 30 min after allowing the mouse to wake up from deep anesthesia (>1% isoflurane, temperature control set to 34°C, blue). Theta, beta, and gamma activity bands arise as the mouse wakes up. Each trace is the average of 30 recordings, 11-second each, at a depth of 40 μm below dura.

These oscillations disappear when the mouse is under high anesthesia with little to no heat control (Fig. 13). Using a single mouse, in a single recording session, oscillations at all points other than a sharp theta band were entirely suppressed under deep anesthesia. The mouse was then allowed to wake up over 20 min, and the recordings were repeated. In the awake recordings, oscillations were detected at ∼35, 25, and 8–10 Hz (Fig. 13).

**Figure 14.**
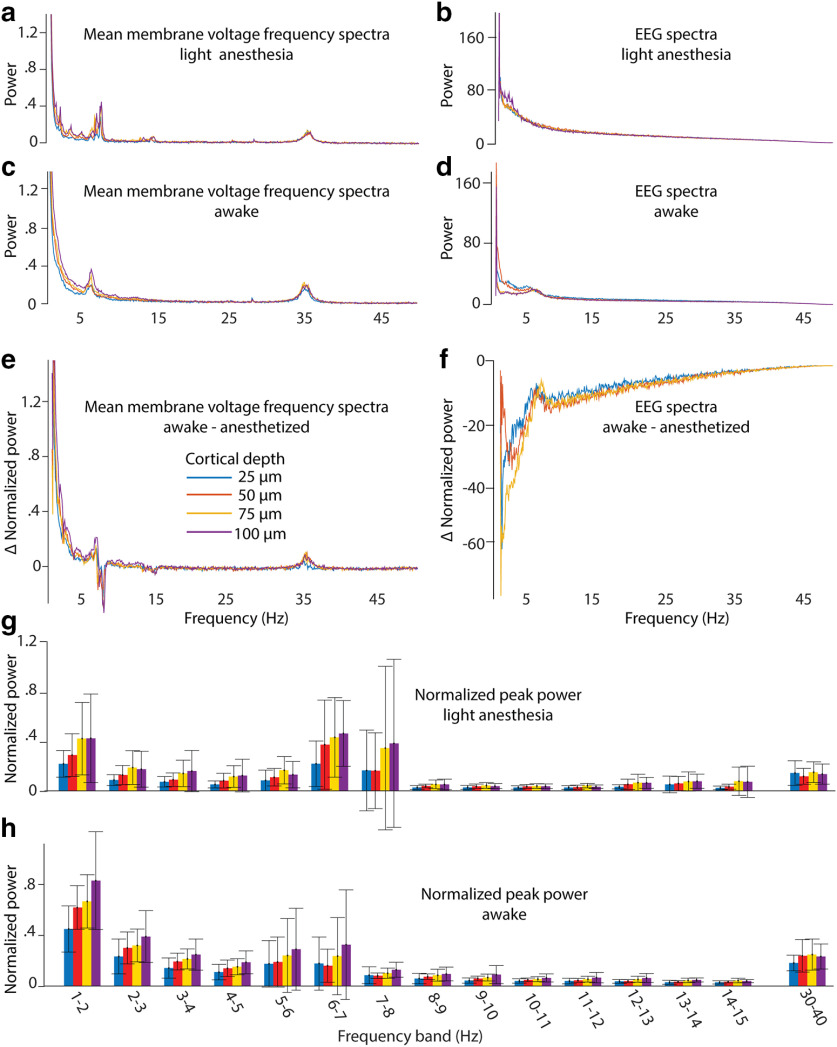
Average frequency spectra. ***a–f***, Average membrane voltage and EEG frequency spectra for awake and lightly anesthetized conditions, separated by cortical depth (210 averages, 7 mice; ***a–d***), and difference between awake and anesthetized spectra (***e***, ***f***). ***g***, ***h***, Small frequency bands were examined, and the maximum power was plotted for anesthetized (***g***) and awake (***h***) spectra. Colors indicate cortical depth; error bars show the SD (7 mice).

To further reduce noise and generalize, we normalized the power spectra and averaged the spectra recorded in different mice (*n* = 7) at four cortical depths in L1, that are 25, 50, 75, and 100 μm below the dura, under light anesthesia and in the awake state and compare them with the corresponding EEG spectra (Fig. 14*a–d*). The differences between the spectra of awake and anesthetized recordings at different cortical depths (Fig. 14*e*,*f*) were largest at <5 Hz. When the animal wakes up, the frequency band at 7 Hz disappears and another at 6 Hz appears. Also, a weak frequency band at 15 Hz disappears when the animal wakes up. As expected, the frequency band at ∼35 Hz is stronger in the awake animals.

To compare the peak power at different cortical depth on a fine frequency scale, we plotted the peak power of 1-Hz frequency bands of every cortical depth as bar graphs (Fig. 14*g*,*h*). As a general trend, the peak power of membrane oscillations increases with cortical depth from 25 to 100 μm in the frequency range of <8 Hz in slightly anesthetized animals and of 7 Hz in awake animals. Interestingly, the 30–40 Hz peak power was constant in respect to cortical depth in the slightly anesthetized mouse as well as in the awake mouse.

**Figure 15.**
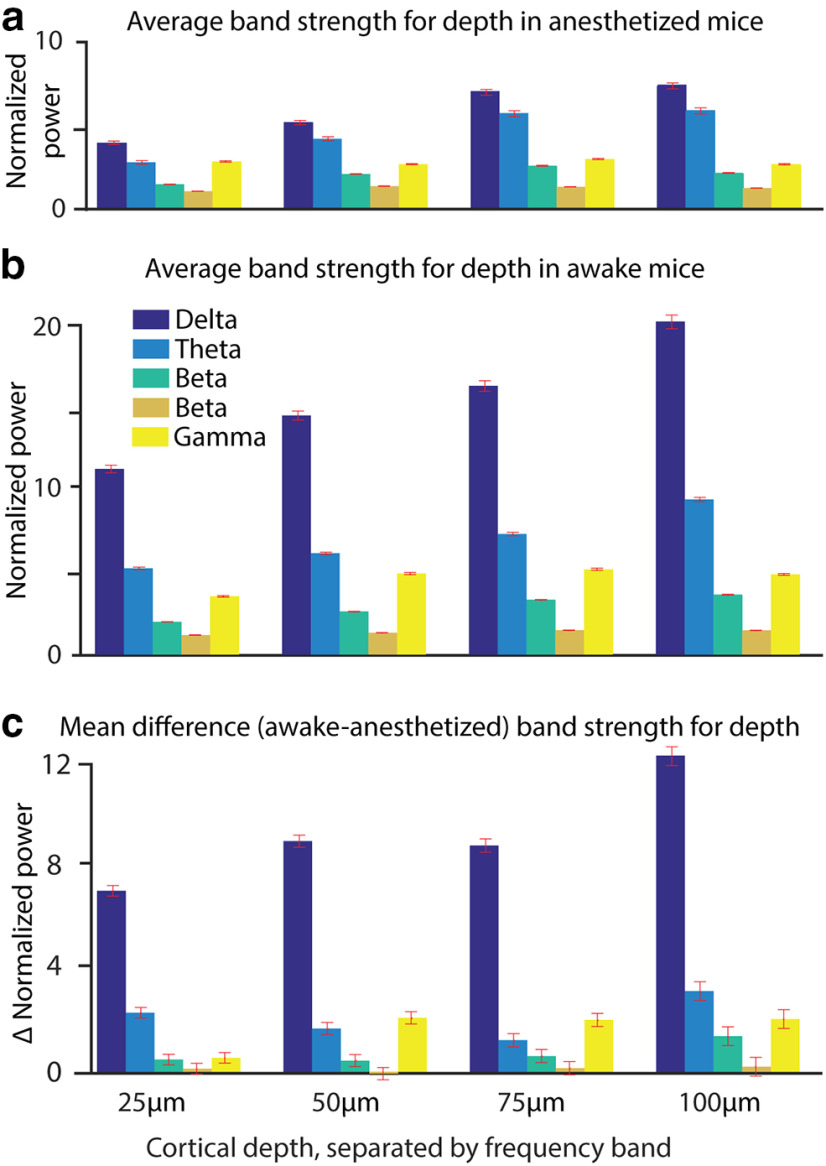
***a***, ***b***, Normalized power of frequency bands delta (0.5–4 Hz), theta (4–10 Hz), lower beta (10–20 Hz), upper beta (20–30 Hz), and lower gamma (30–40 Hz) during light anesthesia (***a***) and wakefulness (***b***) over cortical depth. ***c***, The difference between wakefulness and light anesthesia was calculated within each mouse, and then averaged. Error bars show the SD (30 trials for each of six mice at each depth, so *n* = 360).

Finally, we integrated the spectral power in the canonical oscillation bands of the delta (0.5–4 Hz), theta (4–10 Hz), lower beta (10–20 Hz), higher beta (20–30 Hz), and lower gamma (30–40 Hz) oscillations (Fig. 15). We find a power increase with depth for delta, theta, and lower beta oscillations, and an increase of power in these three bands when switching from light anesthesia to wakefulness. We cannot find spectral power changes in higher beta oscillations. Lower gamma power is constant in slightly anesthetized mice in L1 from 25 to 100 μm. In the awake animal, the spectral power of the lower gamma band increases at 50, 75, and 100 μm, but stays rather constant at 25 μm cortical depth.

## Discussion

di1-ANNINE-6plus is a new version of ANNINE dyes with the same voltage-sensing characteristics which shows great promise for use in L1 voltage imaging. It might also be useful for bath application to label brain slices as di1-ANNINE-6plus diffuses into tissue at least to a depth of 150 μm in 40 min. Other ANNINE-6 variants penetrate tissue only tens of micrometers.

Bubble surgeries are ideal for applying dye to the surface of the brain and achieving L1 staining. This surgery is useful for the application of dyes or drugs that have to be applied in large quantities, and where injections into the tissue would cause damage. Additionally, it avoids the removal of the dura, and therefore reduces invasiveness. We did not find any side effects of H_2_O_2_ application to the dura and O_2_ generation under the dura, as described before when used to remove the dura mater in mouse surgeries (Sheroziya and Timofeev, 2014). Sheroziya and Timofeev (2014) found that peroxide for dura removal was the gentlest method that was tested. Manual dura removal destroyed the long-range, low-frequency oscillations that travel through cortical L1. Dura removal by peroxide application, however, left these connections intact, and thus the oscillations were present. In testing the bubble surgery in our work, pyramidal cell fibers in L1 were left intact, showing calcium activity as close as 15 μm below the cortical surface. di1-ANNINE-6plus applied to the cortical surface provides an interesting new approach to study cortical oscillation within cortical L1. It was possible to record line scans in L1 from multiple points inside the cranial window, up to 3 mm apart. So, large spatial scale recordings with high spatial and temporal resolution are possible with two-photon excitation. Camera imaging should also be possible and will allow L1-specific imaging as the labeling is dominated by the most superficial 50–100 μm.

To confirm the biological origin of the oscillations recorded, a mouse was deeply anesthetized to a point where oscillations and brain activity were dampened. Under this state, all oscillation peaks were dampened or entirely absent. Allowing this mouse to wake up partially (the mouse was not yet running), returned gamma and beta oscillations, and altered the theta and delta oscillations, over the course of 20 min. As the imaging system was not altered during this recording session, and the only change was the state of the animal, the altered oscillations can only be biologically derived (Fig. 13).

We showed that spindle activity can be imaged with di1-ANNINE-6plus and simultaneously be recorded electrically (Fig. 9*g*). However, the frequency spectra of the imaging data and the electrical recording show different features (Fig. 9*h–k*). The reason is the different origin of both signals. The voltage-sensitive dye measures the average membrane depolarization. The EEG is the result of current sources and sinks. In the simplest case of a single depolarizing soma during an action potential, the voltage-sensitive dye would report the membrane depolarization. An extracellular recording would show a biphasic signal of a current sink and then a source. In other words, the EEG signal is proportional to the first derivative of the membrane depolarization. So, the frequency spectra are expected to be different, and in a first approximation the EEG frequency band should be at twice the frequency as that of the voltage imaging. However, the mixing of signals (many processes contribute to the imaging signal; even more structures contribute to the EEG signal), the mixing of different populations of neurons (the imaging signal more local, the EEG averages the signal over several mm), and filtering complicate the relation between voltage imaging frequency spectra and EEG frequency spectra, and further investigation is needed.

Our data show changes in oscillations with depth through L1. Of particular note is the frequency band centered at 35 Hz, corresponding to low gamma oscillation. Gamma in cortex is thought to be created from an interaction between pyramidal neurons and fast-spiking interneurons (Buhl et al., 1998; Bartos et al., 2007; Merker, 2016). L1 is not thought to contain fast-spiking interneurons, although several fast-spiking interneuron types have projections into L1 (Jiang et al., 2015). The majority of neurons found in L1 are slow-spiking neuroglioform cells and single bouquet-like cells. Neither population is thought to be able to generate gamma activity. Additionally, Martinotti cells from both layers 2/3 and 5 have extensive axonal projections in L1, but again do not produce the fast firing necessary for gamma generation. It is possible that a small population of fast-spiking interneurons exists in L1 (Wozny and Williams, 2011), although they are most likely chandelier cells, rather than the basket cells required for gamma generation (Bartos et al., 2007; Buzsáki and Wang, 2012). The only remaining option, if gamma oscillation is generated in L1, is that low gamma oscillations are generated through layer 2 basket cell axon projections (Jiang et al., 2015) onto primary dendrites of pyramidal neurons. Theory suggests gamma generation requires basket cell inhibition onto pyramidal cells, close to the pyramidal cell soma (Buzsáki and Wang, 2012), but pyramidal cell somas are absent >100 μm deep.

There was an increase in the power of delta, theta, and lower beta bands with cortical depth. Increasing power with depth was the expected result of optical voltage recordings in L1 (Kuhn et al., 2008), as anatomy shows a lot of branching, especially in the very superficial range (Cauller et al., 1998; Porrero et al., 2009; Narayanan et al., 2015). The finer and more diverse the processes are, the more different the information they should carry. Therefore, the power of oscillations should decrease closer to the brain surface as signals decorrelate. However, gamma does not experience the same trend, except at 25 μm cortical depth in the awake state, but is rather constant throughout L1.

To estimate the average membrane depolarization, the gamma band (30–40 Hz) was isolated for each cortical depth and brain state (lightly anesthetized and awake), using an inverse fast Fourier transform, over the 30–40 Hz band, with phase removed. This calculates the highest Δ*F*/*F* value for the averaged gamma band (Table 1). If we assume that the recorded gamma oscillations mainly originated from dendritic membrane potential oscillations, we can estimate the average voltage amplitude. Dendrites in the upper cortex make up 23% of the cortical surface area (Williams et al., 1980; Braitenberg and Schüz, 1998). If we assume the same sensitivity of di1-ANNINE-6plus and di4-ANNINE-6plus, the expected sensitivity is −0.47% change/mV when excited at 1020 nm (Kuhn et al., 2004). Using −0.47% change/mV, the gamma oscillations range from 0.09 to 0.14 mV. However, if we assume that not all of the cortex being recorded consisted of dendrites, as explained previously, these oscillations are in the 0.5 mV range (Table 1). This is in line with previously reported gamma oscillation magnitudes (Penttonen et al., 1998).

**Table 1:** Gamma oscillation peak in Δ*F*/*F*, corresponding to the average voltage change if a conversion factor of −0.47%/mV (Kuhn et al., 2004) is applied, and the voltage change under the assumption that only dendrites participate and that 23% of the membrane surface is contributed by dendrites (Braitenberg and Schüz, 1998)

Cortical depth	Light anesthesia			Awake		
	Δ*F*/*F* (%)	Average voltage change (mV)	Dendritic voltage change (mV)	Δ*F*/*F* (%)	Average voltage change (mV)	Dendritic voltage change (mV)
25 μm	0.048	0.10	0.44	0.053	0.11	0.49
50 μm	0.044	0.092	0.40	0.067	0.14	0.62
75 μm	0.045	0.097	0.2	0.057	0.12	0.53
100 μm	0.045	0.095	0.41	0.061	0.13	0.56

The existence of membrane potential oscillations, especially of low gamma, in the distal dendritic tufts of pyramidal neurons identifies L1 as an important coordination hub of top-down input, allowing dynamic binding of neurons for cortical associations (Larkum, 2013).

## Synthesis

Reviewing Editor: Dritan Agalliu, Columbia University Medical Center Decisions are customarily a result of the Reviewing Editor and the peer reviewers coming together and discussing their recommendations until a consensus is reached. When revisions are invited, a fact-based synthesis statement explaining their decision and outlining what is needed to prepare a revision will be listed below. The following reviewer(s) agreed to reveal their identity: Sunil Gandhi.

This manuscript represents a valuable addition to our growing toolkit of voltage sensors. The authors have addressed some of the concerns in the revised manuscript. However, one of the reviewers (Reviewer # 2) is still concerned about the assessment of brain inflammation after the procedure. The authors need to address the concerns of this reviewer in their revised manuscript. Below I provide the comments from each reviewer.

Reviewer # 1 (Minor concerns)

1. Fig 11g: please specify band pass filter used

2. Fig 13: please specify time elapses between the two scans

3. Fig 14: please specify criteria used for running vs. still epochs. How was running measured and threshold used? Please specify here or in methods

4. Line 376: please specify previously reported sensitivity for di4-ANNINE-6 and why the new dye’s sensitivity is lower than the older dye

5. Line 384: correct grammar “This increased penetration”

6. Line 498: reference to Fig 15 instead of 12

Reviewer # 2 (Major concern)

The authors still fail to properly address the concern this reviewer had with respect to the impact of peroxide use during the “bubbling” surgical procedure. Instead of showing the immune response associated with this procedure, the authors have performed calcium imaging on L1. This is not the correct way to show that there is no long term inflammation associated with this rather invasive procedure. The presented calcium data do not allow for a comparison with pre-surgical conditions. Moreover, there are no statistics to compare between the two states. The authors show a few traces (arbitrarily selected) which further raise concern. At present this issue remains of high concern to this reviewer and needs to be addressed in a revised manucript.
